# Enhancing Sensitivity of Point-of-Care Thyroid Diagnosis via Computational Analysis of Lateral Flow Assay Images Using Novel Textural Features and Hybrid-AI Models

**DOI:** 10.3390/bios14120611

**Published:** 2024-12-13

**Authors:** Towfeeq Fairooz, Sara E. McNamee, Dewar Finlay, Kok Yew Ng, James McLaughlin

**Affiliations:** School of Engineering, Ulster University, Belfast BT15 1ED, UK; se.mcnamee@ulster.ac.uk (S.E.M.); d.finlay@ulster.ac.uk (D.F.); mark.ng@ulster.ac.uk (K.Y.N.); jad.mclaughlin@ulster.ac.uk (J.M.)

**Keywords:** convolutional neural network (CNN), lateral flow assay (LFA), point-of-care (POC), gray-level co-occurrence matrix (GLCM), region of interest (ROI), texture analysis, thyroid-stimulating hormone (TSH)

## Abstract

Lateral flow assays are widely used in point-of-care diagnostics but face challenges in sensitivity and accuracy when detecting low analyte concentrations, such as thyroid-stimulating hormone biomarkers. This study aims to enhance assay performance by leveraging textural features and hybrid artificial intelligence models. A modified Gray-Level Co-occurrence Matrix, termed the Averaged Horizontal Multiple Offsets Gray-Level Co-occurrence Matrix, was utilised to compute the textural features of the biosensor assay images. Significant textural features were selected for further analysis. A deep learning Convolutional Neural Network model was employed to extract features from these textural features. Both traditional machine learning models and hybrid artificial intelligence models, which combine Convolutional Neural Network features with traditional algorithms, were used to categorise these textural features based on the thyroid-stimulating hormone concentration levels. The proposed method achieved accuracy levels exceeding 95%. This pioneering study highlights the utility of textural aspects of assay images for accurate predictive disease modelling, offering promising advancements in diagnostics and management within biomedical research.

## 1. Introduction

The prevalence of cardiovascular diseases (CVDs) as the leading global cause of mortality underscores the urgency in understanding their multifaceted pathogenesis [1]. Among the contributing factors, thyroid disorders, encompassing hypothyroidism, hyperthyroidism, and Hashimoto’s thyroiditis, have emerged as significant determinants of cardiovascular health [2]. The regulatory role of thyroid hormones T3 and T4 in lipid metabolism, insulin resistance, and vascular function underscores their profound impact on cardiovascular physiology [2]. Subtle variations in thyroid status within normal ranges can significantly influence the progression of CVDs [3]. Thyroid-Stimulating Hormone (TSH) is crucial for the proper functioning of the neurological system, skeleton, and reproductive system. Additionally, it plays a role in maintaining body weight, cholesterol levels, body temperature, and heart rate [4,5]. Moreover, TSH is pivotal in metabolism regulation as it is closely linked to cardiovascular health, with abnormal levels (0.4–4.5 mIU/L) indicating heightened cardiovascular risk [6]. Hyperthyroidism (TSH < 0.4 mIU/L) and hypothyroidism (TSH > 4.5 mIU/L) are associated with adverse cardiac effects and increased susceptibility to CVDs [7,8]. This highlights the importance of accurate and timely diagnosis of thyroid disorders, particularly when considering their potential contribution to CVDs alongside other health issues, including high cholesterol, infertility, low bone density, muscle weakness, osteoporosis, lupus, and other autoimmune disorders [9,10,11].

The traditional diagnostic methods for thyroid disorders, such as TSH blood testing, often have limitations in terms of cost, time, and accessibility, especially in resource-limited settings [12,13,14]. Point-of-care (POC) testing devices present a promising alternative, offering the advantage of rapid TSH testing without the need for advanced laboratory equipment, thus potentially transforming the diagnosis and monitoring of thyroid disorders in both well-resourced and resource-limited environments [13,15].

Lateral Flow Assays (LFAs), a prominent class of POC biosensors, enable quick and reliable TSH measurements [16,17]. Their simplicity and affordability make LFAs an ideal candidate for widespread use in POC settings, facilitating early detection and timely management of thyroid dysfunction and the cardiovascular disease (CVD) risks associated with thyroid health [16].

### 1.1. Recent Advances in LFA Technology

In recent years, LFAs have undergone significant advancements, particularly in improving their analytical performance through innovative material use and signal amplification techniques. This is largely due to the versatility, affordability, and biodegradability of LFAs compared to other biosensor types, such as chip-based or textile-based platforms. For example, paper-based LFAs are widely adopted for their sustainability and cost-effectiveness, and they are now commonly used for detecting infectious diseases, environmental pollutants, and food safety markers [18].

To enhance detection sensitivity, researchers have incorporated nanomaterials with specialised detection properties into LFAs, enabling modalities such as fluorescence, Raman scattering, thermal detection, and magnetic detection [19,20,21,22]. The integration of multiple detection modalities has led to the development of multimodal LFAs, which combine colorimetric, magnetic, and Raman signals to achieve high-sensitivity detection of biomarkers like human chorionic gonadotropin (HCG) [22,23]. Such innovations are particularly useful for applications requiring high specificity and sensitivity, enabling more precise biomarker quantification.

**Figure 1:**
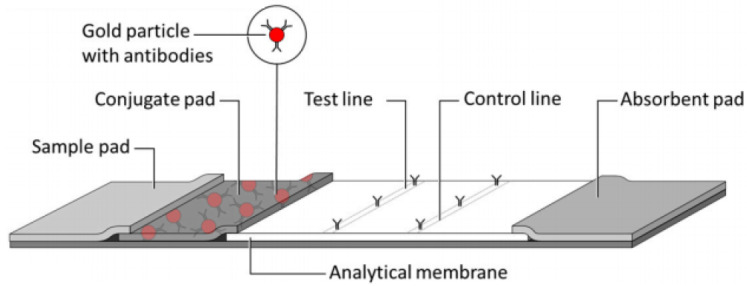
Typical structure of an LFA (taken from [17]).

The typical LFA structure, as shown in Figure 1, includes components such as a sample pad, conjugate pad, nitrocellulose membrane, and absorbent pads. In this structure, the analyte biomarkers within the sample interact with nanoparticles immobilised on the membrane, resulting in color changes in the test line. The intensity of the color correlates with the concentration of the target analyte [24].

### 1.2. Applications of LFAs in POC Diagnostics

LFAs have a rich history in diagnostic testing, initially introduced during the latex agglutination test by Singer and Plotz [25]. Today, they are widely applied for detecting infectious diseases, such as in COVID-19 antibody tests, the influenza A (H1N1) test, as well as in home pregnancy tests [26,27,28,29]. LFAs are also used in environmental monitoring, including pesticide detection [30] and food contamination analysis [31].

POC testing has been particularly valuable in resource-limited settings. Notable examples include diagnostic tests for tetanus immunity in Iran, glucose-level testing for diabetes in rural India, hypoglycemia detection in Nigerian children, and trypanosomiasis diagnosis in Angola [32]. These applications illustrate the growing reliance on LFAs as cost-effective and accessible diagnostic tools across diverse healthcare settings.

### 1.3. Challenges and Sensitivity Enhancement in LFA Diagnostics

Despite their advantages, LFAs still face challenges in achieving high sensitivity and specificity. Variations in color perception, lighting, and orientation can lead to inconsistencies, particularly at low analyte concentrations [33]. Visual interpretation, the most common method for analyzing LFA results, is inherently subjective and varies with individual perception. For example, one in twelve men worldwide are affected by color blindness, impacting their ability to interpret LFA results accurately, especially in distinguishing shades of red, yellow, and green [33].

To address these limitations, researchers have developed various sensitivity enhancement techniques. These include probe-based signal amplification [34], temperature–humidity adjustments [35], and fluidic control methods [36]. However, these approaches often involve complex or costly materials and equipment, limiting their scalability and practicality. The recent advancements also involve LFA manufacturing optimisation [37] and specialised devices for LFA result reading, such as CCD cameras, magnetic assay readers, and LED excitation-based readers [38,39].

In this study, gold nanoparticles in LFAs are leveraged for colorimetric detection, a preferred approach due to its compatibility with low-cost, accessible imaging devices like smartphones and CMOS cameras. The visual interpretation challenges are mitigated through digital analysis of LFA test lines and control lines using image processing tools that analyse colour intensities captured by CMOS cameras. For instance, CMOS-based systems have been successfully used for cortisol-level measurement in human saliva [40] and other types of analyte detection in LFAs [41,42,43]. These studies, along with the recent work by Fairooz et al. [44], employ intensity-based quantification methods, although these remain susceptible to variations caused by lighting and background differences.

## 2. Textural-Analysis-Based Sensitivity Enhancement

While intensity-based methods are widely used, they are often inadequate for reliable LFA quantification due to environmental inconsistencies. Instead, textural analysis offers a more robust approach by examining the gray-level spatial distribution patterns within LFA images, providing a quantitative description of the image content beyond simple pixel intensity values [45]. This enables improved classification accuracy under varying lighting and background conditions, making textural analysis a valuable tool for enhancing LFA sensitivity and reliability.

This study introduces a novel approach to enhance LFA sensitivity by utilising the Gray-Level Co-occurrence Matrix (GLCM) [45], a statistical technique commonly employed in medical imaging, to capture spatial dependencies within LFA images. While previous studies have analysed entire LFA strip images, including non-diagnostic background regions [46], this research with a custom implementation, averaged horizontal multi-offset GLCM (AHMO-GLCM), is designed specifically for extracting textural features from the diagnostic ROIs on LFA strips, such as test and control lines, while minimising background noise. Unlike the conventional methods, this approach focuses on textural rather than intensity-based features, offering improved sensitivity and reliability, particularly under variable lighting conditions.

By isolating diagnostic ROIs and employing classifiers such as CNN–SVM and Wide Neural Networks, the proposed method achieves robust quantification of TSH levels across different concentrations. The approach is particularly advantageous in low-resource settings where precise environmental controls are challenging, thus enabling broader accessibility to sensitive diagnostic tools. The enhanced sensitivity and reliability offered by our AHMO-GLCM-based computational method mark a significant advancement over the traditional visual or pixel-intensity-based approaches.

### Dataset and LFA Preparation

This study utilised LFA strip images specifically produced in-lab to detect varying concentrations of TSH. The preparation involved several key steps, including the formulation of the test and control lines, the conjugation of the detection antibody, and the assembly of the LFA strips.

The TSH capture antibody and control line antibody were initially prepared by diluting them to a concentration of 2 mg/mL in a printing buffer composed of 10 mM PBS and 1% sucrose, with a pH of 7.4. These test and control antibodies were then printed onto a 25 mm nitrocellulose membrane (Sartorius CN95, from the company Sartorius AG, which is originally from Göttingen, Germany) using a BioDot XZ1010 printer (BioDot Inc., Irvine, CA, USA) at a controlled flow rate of 1 μL/cm.

To prepare the conjugated TSH detection antibody, gold nanoparticles (40 nm, OD40) were conjugated to the TSH detection antibody using a modified protocol adapted from Abcam. In this process, 50 μL of gold nanoparticles, 10 μL of 150 mM MES buffer (pH 5.0), 20 μL of TSH detection antibody at a concentration of 500 μg/mL, 20 μL of 1 mM EDC, and 40 μL of 1 mM sulfo-NHS were mixed together. The solution was incubated at room temperature on a roller for 30 min. To halt the reaction and stabilise the conjugate, 0.5 μL of hydroxylamine was added, followed by a further 10-minute incubation. The gold nanoparticles were functionalised with a carboxyl group, which is used to covalently bind the TSH antibody using EDC/NHS chemistry. The hydroxylamine used acted as a blocking agent, helping to prevent non-specific binding and ensure accurate detection by maintaining the antibody’s specific binding sites. Hydroxylamine generally acts by reducing potential cross-reactivity sites without affecting the functional epitopes of the antibodies.

Following the conjugation, post-processing involved adding 1 mL of TBS (50 mM, pH 8.5) containing 0.05% Tween-20 to the conjugate mixture. The solution was then centrifuged at 10,000 rpm for 10 min at 20 °C. After centrifugation, the supernatant was removed, and the resulting pellet of the gold–TSH–NP was re-suspended in 400 μL of gold drying buffer, a solution of TBS (50 mM), 0.5% BSA, 0.05% Tween-20, and 2% sucrose. This prepared gold–TSH–NP was then sprayed onto a 17 mm glass fiber conjugate pad (Ahlstrom, the company is originally from Helsinki, Finland) using the BioDot XZ1010 printer (BioDot Inc., Irvine, CA, USA) at a rate of μL/cm.

For the assembly of the LFA strips, the printed nitrocellulose membrane, along with the prepared conjugate pad, an absorbent pad (45 mm, Ahlstrom, A238), and a sample pad (10 mm, Ahlstrom, 1668 HV+), were laminated onto a 0.01-inch-thick plastic backing card (Lohmann, the company loacted in Neuwied, Germany). These laminated components were subsequently cut into 5 mm strips using a guillotine, finalizing the LFA strip assembly.

To run the LFAs, a sample volume of 50 µL and 70 µL of running buffer (10 mM PBS with 2% BSA and 1% Tween-20) were added to a microtitre plate well containing an LFA strip. The strips were allowed to run for 30 min, facilitating the migration of the sample along the strip.

Finally, LFA strips were produced with specific TSH concentration ranges to enable sensitivity testing. These concentrations included 0, 0.5, 1, 2.5, 5, 10, 25, 50, and 100 mIU/L, providing a range from low to high TSH levels. Each strip was analysed both visually and with an LFA reader to evaluate detection across this concentration range.

## 3. Methods

**Figure 2:**
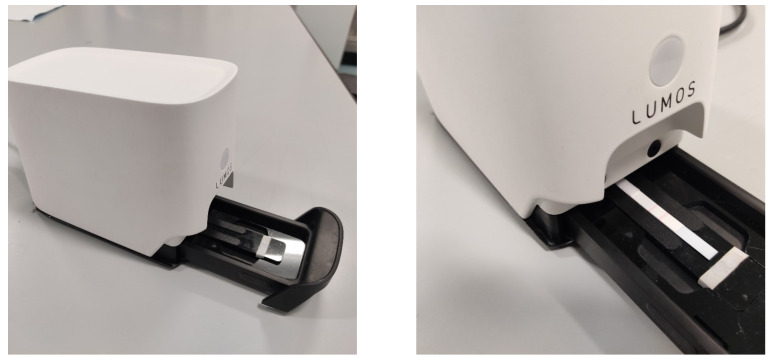
Lumos Reader device for capturing LFA images.

The methodology developed and implemented in this study included the following key stages: LFA image data acquisition, image pre-processing, feature development, feature extraction, and model training and evaluation. The acquisition of LFA image data was carried out using Lumos Leelu LFA Reader from Lumos Diagnostics LTD [47] (Lumos Diagnostic’s US headquarters is in Carlsbad, California, USA), as shown in Figure 2.

**Figure 3:**
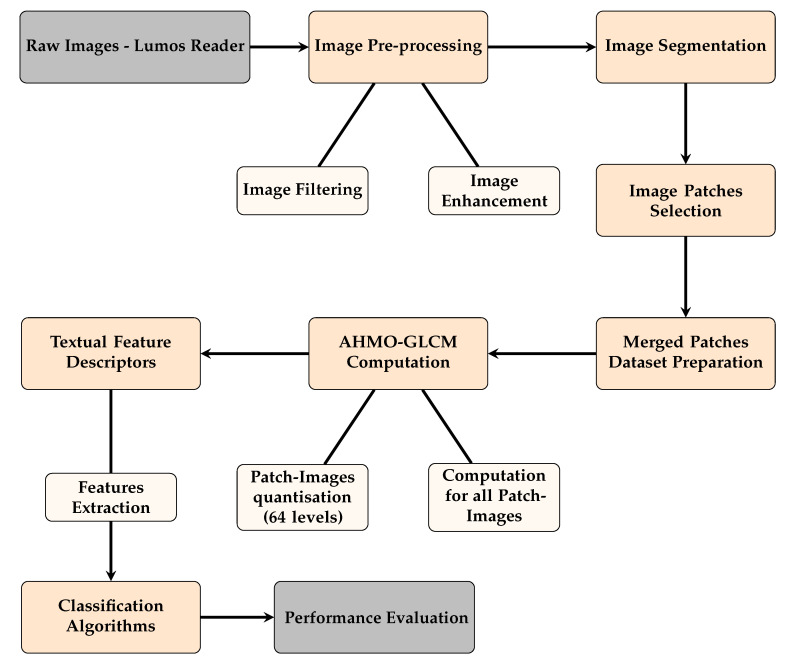
Schematic of the methodology for LFA image analysis using textural features.

The overall methodology implemented, from image capture through the Lumos Leelu Reader to model evaluation, is outlined in the schematic diagram shown in Figure 3.

### 3.1. LFA Image Data Acquisition

High-resolution images of the LFA samples were acquired using the Lumos Reader, which features a high-sensitivity imaging assembly with a precision lens capturing 33 pixel/mm images of test strips up to 50 × 110 mm. Uniform illumination across LFA strip area was provided by an integrated LED array. Through calibrated image capture and software-defined assay-specific settings, the reader facilitated standardised acquisition of high-quality image data. The capabilities of uniform illumination and adjustable imaging parameters enabled reproducible digitisation of LFA test results. These standardised data served as input for subsequent methodology steps involving image processing and analysis.

All TSH LFAs spanning 0.5–100 mIU/L were imaged under controlled, standardised parameters to minimise errors. Images of 700-by-145 pixels were captured, excluding 0 TSH levels. Concentrations considered were 0.5, 1, 2.5, 5, 10, 25, 50, and 100 mIU/L and labelled accordingly.

### 3.2. Image Pre-Processing

The Lumos LFA Reader utilises a meticulous pre-processing protocol to ensure consistent results. Calibration of the reader ensures accurate data, while automated strip insertion minimises errors that may arise due to improper positioning. Further accuracy was achieved through removing electrical noise (dark current) and unwanted light (stray light). Pre-processing extracted key diagnostic regions from the captured images and generated standardised images of dimension 700-by-145 pixels for further analysis. All samples were read 30 min after the assay for consistency. Image sharpening and contrast adjustments were also applied to enhance the standardised images.

### 3.3. Image Segmentation and ROI Detection

Following image pre-processing, accurate segmentation was essential to isolate the diagnostically relevant test and control lines within LFA images, applicable to both colorimetric and fluorescent assays. Thresholding [48], a common segmentation technique, was employed. Here, pixels exceeding a specific intensity threshold were classified as foreground, likely containing the test and control lines, while the remaining regions were considered background.

**Figure 4:**
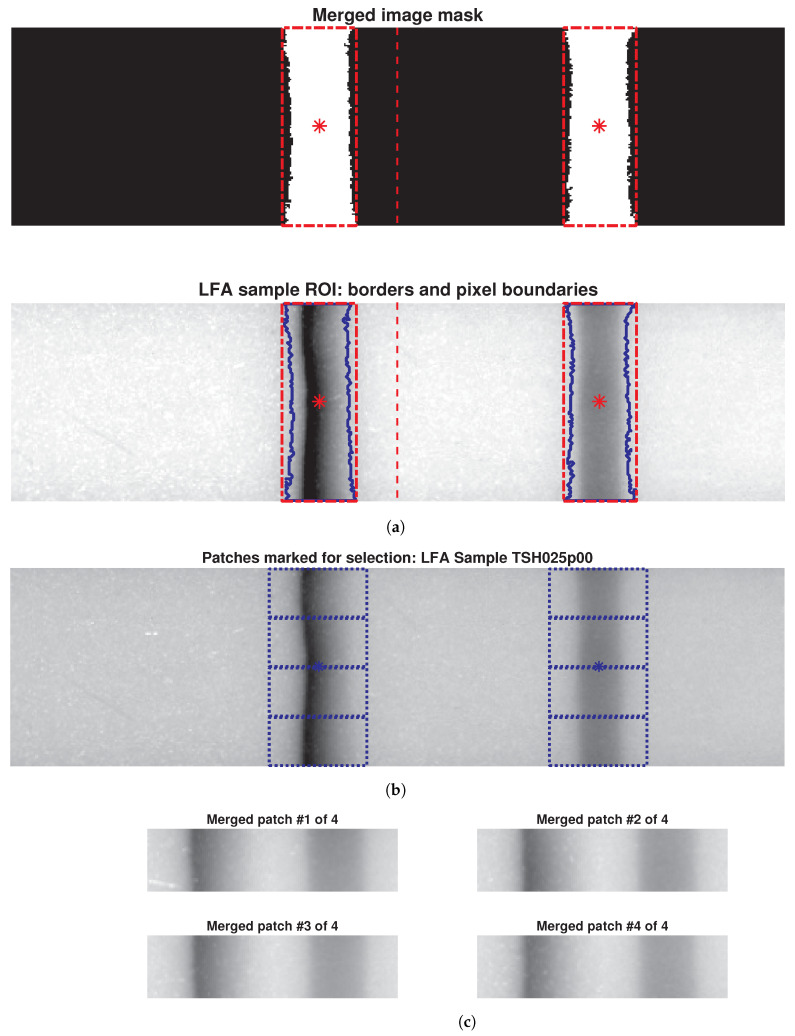
(**a**) Merged binary masks of segmented halves with bounding boxes (red lines) marking the ROIs, and LFA sample overlaid on marked binary masks. (**b**) Patch creation: 500-by-128 pixel LFA image sample; (**c**) four 128-by-32 pixel patches extracted from ROIs and merged.

The originally acquired 700-by-145 pixel images from the reader were downsized to a uniform dimension of 500-by-128 pixels to remove potential scan artifacts and ensure consistency for further analysis. Due to variations in intensity levels within the standardised images, a single global threshold value could not effectively segment both test and control lines simultaneously. To address this limitation, adaptive window selection thresholding [49], capable of handling such intensity variations, was employed by strategically splitting each LFA image into two halves (250-by-128 pixels each) along the vertical axis, as indicated by the red dashed line in Figure 4a. This approach successfully identified the test and control lines within their respective halves.

Following segmentation, the processed image halves and their corresponding binary masks (representing the extracted lines as foreground pixels) were merged together (Figure 4a). Bounding boxes (red) and boundaries (blue) around the test and control lines highlight the ROIs, while the “*” in the images denotes the pixels centroid location of the ROIs. These steps ensure that critical diagnostic information is retained for subsequent analysis.

### 3.4. Patch Selection and Dataset Expansion

Despite pre-processed and segmented images, the limited quantity of LFA samples with varying TSH concentrations hindered accurate machine learning predictions. Large datasets are crucial for AI models to learn complex patterns and features. This prevents overfitting and improves generalisation for unseen samples. Additionally, a larger dataset enables robust model evaluation.

To address this limitation, an expanded dataset was generated from the existing LFA images. A meticulous process involving marking, selecting, and cropping regions within ROIs was conducted to extract informative patches (Figure 4b). These patches, measuring 128-by-32 pixels, captured both textural details and background information from test and control lines (potentially relevant for TSH-level differentiation). Four separate patches were extracted from each original LFA image (Figure 4c), quadrupling the dataset size. Consequently, the initial 100 LFA images per concentration level expanded to 400 patch images, and the entire dataset grew from 800 LFA images to 3200 patch images, a fourfold increase. This approach significantly enriched the dataset for robust deep learning analysis.

### 3.5. Averaged Horizontal Multi-Offset GLCM

This section introduces the averaged horizontal multi-offset GLCM (AHMO-GLCM), a novel method used for efficient LFA analysis. AHMO-GLCM modifies the conventional GLCM approach to achieve more robust texture feature extraction.

**Figure 5:**
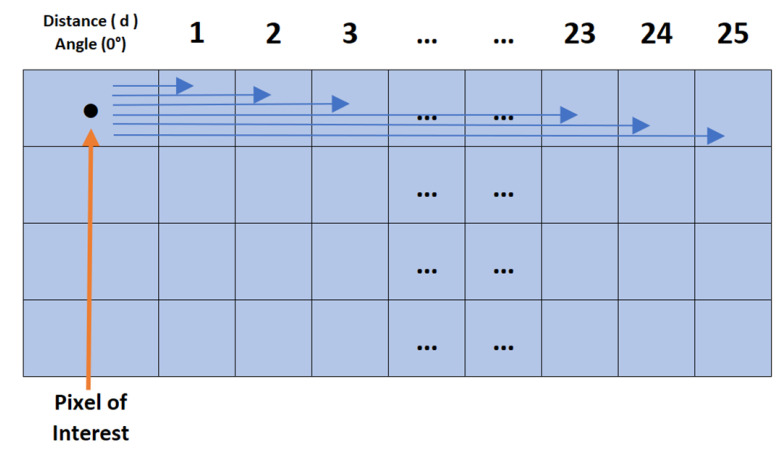
AHMO-GLCM process: GLCM computation at 0 degrees offset with pixel pairs separated by distances d = 1 to 25.

The AHMO-GLCM process (Figure 5) begins by converting colour LFA images to grayscale. This is conducted for consistent pixel intensity analysis as colour variations can significantly impact texture analysis. The images are then quantised.

**Figure 6:**
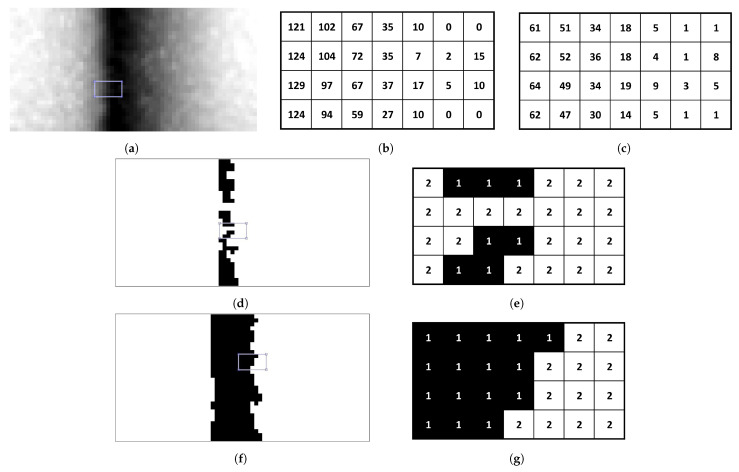
Quantisation: (**a**) split LFA patch and selective sub-patch from test line. (**b**) Pixel values of selected sub-region. (**c**) 64-level quantised sub-patch, capturing intensity variation. Quantised binary patch images: (**d**,**e**) 8-level binarised split patch and sub-patch pixel values. (**f**,**g**) 64-level binarised split patch and sub-patch pixel values.

Traditionally, 8-level quantisation is used for grayscale images. However, this does not effectively capture subtle variations in LFA textures, leading to crucial pixel information loss and hindering accurate classification (Figure 6d; zoomed in Figure 6e). To address this limitation, AHMO-GLCM employs 64-level quantisation (Figure 6f; zoomed in Figure 6g). This captures finer texture details compared to 8-level quantisation, preserving important information for subsequent analysis. To illustrate this, consider a small sub-patch from a grayscale image (Figure 6a). The original pixel intensity values range from 0 to 129 (as shown in Figure 6b). With 64-level quantisation, this range is divided into 64 bins, resulting in quantised intensity levels that capture a wider range of variations. Original values are mapped to their closest quantised level.

After quantisation, GLCM values are computed for each pixel in the quantised grayscale patch image in the horizontal direction for varying offset distances (d = 1 to 25 pixels), capturing texture variations across different spatial scales. To obtain a comprehensive texture measure per pixel, the individual GLCMs are averaged, resulting in a single AHMO-GLCM value summarising textural information across spatial neighbourhoods. Various textural properties are then extracted from the AHMO-GLCM values to quantify different aspects of texture (Figure 6a). These texture features serve as discriminative features for classification.

### 3.6. Texture Feature Extraction and Selection

Following AHMO-GLCM processing, various texture properties were extracted, including contrast, correlation, energy, and homogeneity. To enrich the feature set, additional Haralick GLCM features were derived based on the methods by Uppuluri [50] and Haralick et al. [45]. This resulted in a total of 22 features: autocorrelation (autoc), contrast (contr), correlation (corrm), cluster prominence (cprom), cluster shade (cshad), dissimilarity (dissi), energy (energ), entropy (entro), homogeneity (homom), maximum probability (maxpr), sum of squares: sum average (savgh), sum entropy (senth), sum variance (svarh), difference variance (dvarh), difference entropy (denth), information measure of correlation-1 (inf1h), information measure of correlation-2 (inf2h), inverse difference normalised (indnc), and inverse difference moment normalised (idmnc).

**Figure 7:**
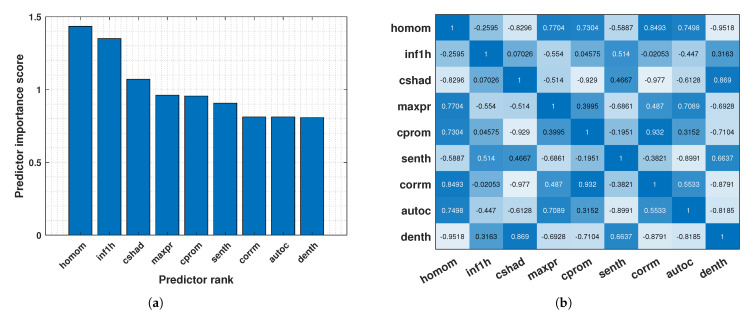
(**a**) Ranking of key texture features based on AHMO−GLCM properties. (**b**) Heatmap of AHMO−GLCM feature correlations.

However, using many features can negatively impact model performance. To address this, a feature selection technique called Maximum Relevance Minimum Redundancy (MRMR) was employed [51]. MRMR selects the most relevant and non-redundant features for classification. In this case, MRMR identified the top 9 most significant features from the original set of 22 (Figure 7a). The correlation heatmap of these top features is visualised in Figure 7b.

Homogeneity (homom):

(1) $$homom=\sum_{i=1}^{N_{g}}\sum_{j=1}^{N_{g}}\frac{p(i,j)}{1+|i-j|}$$

Inverse difference normalised (INN) (indnc):

(2) $$indnc=\sum_{i=1}^{N_{g}}\sum_{j=1}^{N_{g}}\frac{p(i,j)}{1+{\left(i-j\right)}^{2}}$$

Cluster shade (cshad):

(3) $$cshad=\sum_{i=1}^{N_{g}}\sum_{j=1}^{N_{g}}{\left(i+j-\mu_{x}-\mu_{y}\right)}^{3}p\left(i,j\right);$$

Maximum probability (maxpr):

(4) maxpr=max(max(p(i,j)));

Cluster prominence (cprom):

(5) $$cshad=\sum_{i=1}^{N_{g}}\sum_{j=1}^{N_{g}}{\left(i+j-\mu_{x}-\mu_{y}\right)}^{4}p\left(i,j\right);$$

Sum entropy (senth):

(6) $$senth=-\sum_{k=2}^{2N_{g}}p_{X+Y}\left(k\right)\log_{2}\left(\left[p_{X+Y}\left(k\right)\right]+\epsilon \right);$$

Correlation (corrm):

(7) $$corrm=\frac{\sum_{i=1}^{N_{g}}\sum_{j=1}^{N_{g}}\left(i-\mu_{x}\right)\left(j-\mu_{y}\right)p\left(i,j\right)}{\sigma_{x}\sigma_{y}}$$

Autocorrelation (autoc):

(8) $$autoc=\sum_{i=1}^{N_{g}}\sum_{j=1}^{N_{g}}\left(ij\right)p\left(i,j\right)$$

Difference entropy (denth):

(9) $$denth=-\sum_{k=2}^{2N_{g}}p_{X-Y}\left(k\right)\log_{2}\left(\left[p_{X-Y}\left(k\right)\right]+\epsilon \right);$$

GLCM Features Notation:

$N_{g}$: image grayscale levels (e.g., 256 for 8-bit grayscale);

p(i,j): normalised GLCM value (position (i,j) in the matrix);

$p_{X+Y}\left(k\right)$: sum probability distribution (sum of normalised values where i+j=k);

$\mu_{x}$, $\mu_{y}$ (rows), $\sigma_{x}$, $\sigma_{y}$ (columns): marginal means and standard deviations;

ϵ (epsilon): small constant to prevent zero-logarithms (used for zero-probability elements).

### 3.7. CNN for Tabular Data

While typically used for images, Convolutional Neural Networks (CNNs) can also handle other data types as multidimensional tensors. In this case, the 22 AHMO-GLCM features for each sample were reshaped into a 2D “image” of size (22, 1, 1). This format enables the CNN to effectively process and extract features for classification tasks. The developed 2D CNN model utilised three convolutional layers. Each convolutional layer employed 3 × 3 filters with “same” padding, ensuring the output feature maps maintained the same spatial dimensions as the input. This padding strategy is crucial to avoid losing information at the edges of the data.

Following each convolutional layer, batch normalisation was applied to stabilise the learning process and improve training efficiency. ReLU (Rectified Linear Unit) activation was then used to introduce non-linearity into the network, enabling it to capture more complex relationships within the features. In between convolutional layers, max pooling layers were employed to reduce the data dimensionality. These layers take the maximum value within a specific window (2 × 2 in this case) and move the window with a stride of 2 (skipping one element) while maintaining some spatial information. Padding of [0, 1, 0, 1] ensured that the output dimensions remained compatible with subsequent layers.

Finally, the output from the last convolutional layer was fed into a fully connected layer with 8 neurons. This layer transforms the extracted features into a single output vector representing the likelihood of each class. The SoftMax activation function was applied at the output layer to normalise these values into probabilities, enabling the model to classify the input data into one of the predefined categories.

### 3.8. CNN Training and Feature Exploration

**Figure 8:**
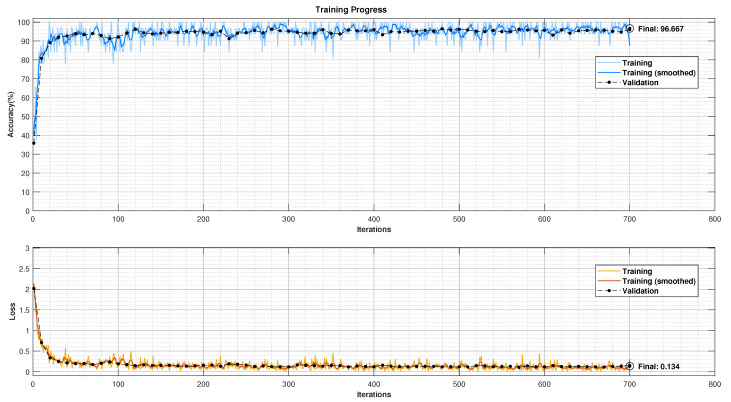
CNN training and validation progress plot (gray images’ bin size: 64; features’ batch processing size: 32).

The CNN model was trained using Stochastic Gradient Descent with Momentum (SGDM) to optimise the model’s performance. Specific training parameters were chosen to balance learning and prevent overfitting. These included a learning rate of 0.01, a maximum of 10 epochs, and a mini-batch size of 32 patch images. Additionally, the data were shuffled before each epoch to expose the model to the training data in different orders, improving robustness. The model’s performance on unseen data was monitored throughout training using a 15% validation set. This validation split helps to identify potential overfitting where the model performs well on training data but poorly on unseen data. The achieved validation accuracy was 96.67%, visualised in Figure 8.

Following successful training, the CNN transformed from a classifier to a powerful feature extractor. By processing the original data (3200 patch images with 22 AHMO-GLCM features), the CNN identified high-level abstract features termed “CNN features”. These features captured complex relationships within the data that might not be readily apparent in the original AHMO-GLCM features.

The study investigated the influence of different feature sets on classification accuracy. To ensure the model encountered a representative mix of data, the data were strategically divided into training (70%), validation (15%), and testing (15%) sets (as supported by Gholamy et al. [52]). Three sets were investigated: the original set containing “All” 22 features, a reduced set containing only the top 9 “MRMR” features, and “CNN feature” extracted by the CNN model itself. Furthermore, to exploit the strengths of both deep learning and traditional machine learning, hybrid models were employed. These models combined the CNN as a feature extractor with established classifiers like SVM, random forest, and decision tree, aiming for even more robust and accurate classification of TSH concentration levels.

### 3.9. Performance Evaluation

**Figure 9:**
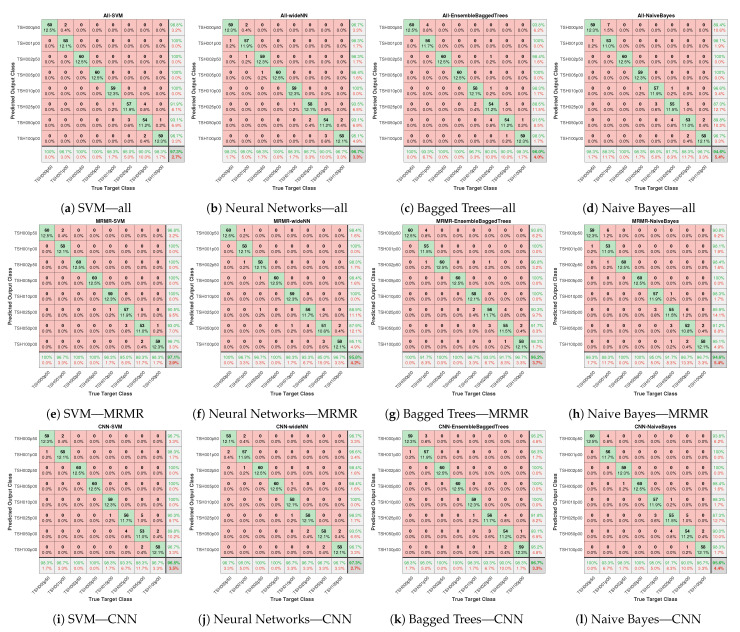
AHMO-GLCM features: confusion matrices comparing classifiers trained on (**a**–**d**) with all features; (**e**–**h**) with MRMR-based top-ranked features; (**i**–**l**) with CNN-derived features.

Figure 9 presents the prediction accuracy and confusion matrices for various classifiers using “All”, “MRMR”, and “CNN” features. Subfigures (a–d) show results across four different classifiers.

The model performance was evaluated on a 15% held-out testing set (stratified across all 8 TSH levels) using sensitivity, specificity, and overall accuracy.

(10) $$Sensitivity\left(\%\right)=\left(\frac{TP}{TP+FN}\right)*100$$

(11) $$Specificity\left(\%\right)=\left(\frac{TN}{TN+FP}\right)*100$$

(12) $$Accuracy\left(\%\right)=\left(\frac{TP+TN}{TP+TN+FP+FN}\right)*100$$

True positives (*TPs*) and true negatives (*TNs*) correspond to the accurately classified patch images. Conversely, false positives (*FPs*) and false negatives (*FNs*) represent the incorrectly classified image patches.

## 4. Results and Discussion

**Figure 10:**
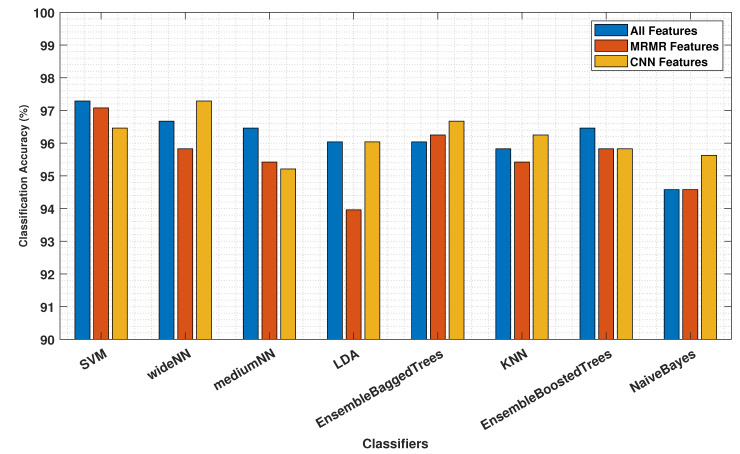
Classification accuracy comparison using all features, top MRMR features, and CNN-derived features from AHMO-GLCM features data.

The proposed approach utilising AHMO-GLCM features for LFA image classification based on TSH concentration levels demonstrated promising results. As shown in Table 1, various machine learning classifiers achieved exceptional accuracy, exceeding 95% on the test dataset across different feature sets. Notably, the CNN-extracted features achieved the highest overall accuracy (97.29%) compared to both “All” features (96.67%) and “MRMR” features (95.83%). Figure 10 also suggests that the CNN features captured more discriminative information for LFA classification. Interestingly, “MRMR” features still outperformed “All” features (96.25% vs. 96.04%), indicating some redundancy within the original set. Additionally, the strong specificity (>99%) by all the models and all the feature sets further highlights the method’s robustness in correctly identifying true negatives. This finding suggests that machine learning, coupled with appropriate feature extraction methods like AHMO-GLCM, has the potential to play a significant role in LFA image analysis for medical diagnostics.

Traditionally, intensity-based methods have been employed for LFA image analysis. However, these methods often struggle to capture subtle variations within the images that may be crucial for accurate diagnoses. AHMO-GLCM offers a significant advantage by analysing the textural features in addition to intensity. This ability to capture the subtle textural variations in LFA images enables more nuanced image analysis, potentially leading to improved diagnostic accuracy.

The high classification accuracy achieved by the proposed approach, particularly with the SVM (>96.96%) and CNN-derived features (>97.29%), suggests its potential to significantly improve the accuracy and efficiency of LFA-based thyroid testing. Early and accurate detection of thyroid disorders like hyperthyroidism and hypothyroidism can have substantial clinical implications. By enabling faster and more reliable diagnoses, AHMO-GLCM could expedite the initiation of appropriate treatment, potentially leading to improved patient outcomes and reduced healthcare costs.

Despite the promising results using AHMO-GLCM features for LFA image classification, limitations exist. The higher dimensionality of these features results in longer processing times, especially with large datasets. Future research aims to enhance the computational efficiency for handling AHMO-GLCM features. Moreover, although this study concentrated on TSH analysis, it is essential to conduct broader evaluations using larger and more diverse LFA datasets, including various biomarkers, to prevent overfitting and bias. Refining the feature selection methods and ensuring generalisability across a wider array of data will be critical for future developments.

## 5. Comparative Analysis of Imaging Techniques in Diagnostic Testing

A direct comparison between the proposed AI-integrated approach for LFA analysis detecting TSH biomarkers and prior studies is challenging due to differing methodologies. However, a contextual comparison highlights the advantages of the proposed textural-features-based method. Previous research, such as the studies by Mutlu et al. [53], Minjing et al. [54], Solmaz et al. [55], and Rahmat et al. [56], has explored digital image analysis and machine learning for interpreting diagnostic tools like pH strips, colorimetric assays, LFAs, and urine dipsticks. These approaches, while innovative, often faced limitations that the proposed method addresses effectively.

Ali Mutlu et al. developed a machine learning approach for colorimetric pH strip detection. However, the absence of standardised illumination conditions during data acquisition impacted the reliability and generalisability of their model. In contrast, the proposed textural features method was developed under strict standardisation protocols, including controlled illumination and noise correction with the image capture within the enclosure box and uniform lighting across the strip surface

**Figure 11:**
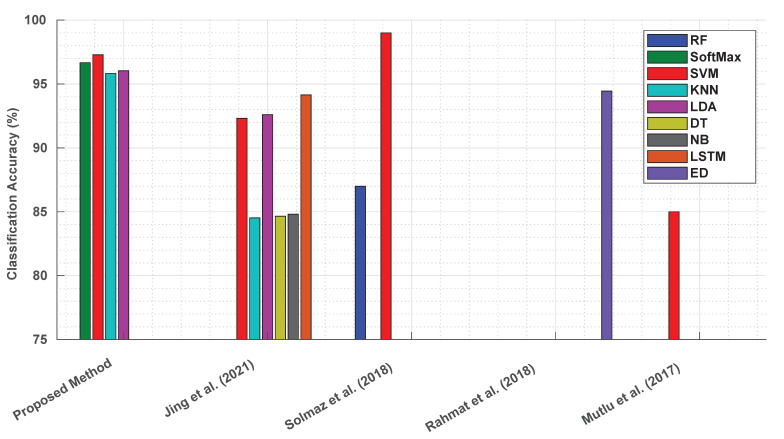
Comparison of classification accuracies: proposed textural features method vs. previous approaches (RF: random forest; LSTM: long short-term memory; ED: Euclidean distance) [53,54,55,56].

Min Jing et al.’s LFA image analysis included irrelevant white membrane background regions, potentially introducing noise and degrading the model accuracy. The proposed method, by focusing exclusively on ROIs and excluding extraneous areas, optimised the data analysis and improved the performance. This precision is reflected in the high classification accuracies achieved, as shown in Figure 11.

Solmaz et al.’s work utilised a limited dataset of 385 smartphone images for pH classification, compromising the robustness and generalisability of their model. Their methodology also lacked standardisation during data collection. The proposed method overcame these challenges by employing a significantly larger dataset, rigorous standardisation during data acquisition, and data processing, resulting in a highly robust classification model.

Similarly, Rahmat et al.’s study on dipstick images faced reproducibility challenges due to insufficient details regarding data acquisition and sample characteristics. The proposed method, in contrast, adhered to a well-defined framework for data acquisition, pre-processing, feature extraction, and model implementation.

The proposed method’s focus on textural features, derived from the multi-offset GLCM, captured the fine-grained relationships between neighbouring pixels, enabling the precise quantification of the biomarker concentrations. Unlike prior intensity-based approaches, this method demonstrated consistent performance, with classification accuracies exceeding 95%, as depicted in Figure 11.

## 6. Conclusions

This study explored the potential of AHMO-GLCM, an innovative texture-based feature extraction method, for classifying TSH levels in LFA images. The findings demonstrated that AHMO-GLCM features are highly effective, with various machine learning classifiers achieving over 95% accuracy in distinguishing LFA images based on TSH concentrations. This method provides a significant advantage over the traditional intensity-based approaches by capturing subtle textural variations within LFA images. The high classification accuracy indicates the potential to enhance the accuracy and efficiency of LFA-based thyroid testing, leading to earlier diagnosis.

The high dimensionality of AHMO-GLCM features and the focus on TSH analysis alone poses limitations, which must be addressed in the future research. By optimising the computational efficiency, refining the feature selection techniques, and expanding the validation, the AHMO-GLCM approach shows promise as a valuable tool for LFA analysis, with broader applications in clinical diagnostics for various health-related biomarkers.

## Figures and Tables

**Table 1:** Performance comparison of ML models using All AHMO-GLCM textural features for LFA images, MRMR selective features, and CNN-derived special features derived from AHMO-GLCM data (image batch size: 64).

Classifiers		Accuracy (%)			Sensitivity (%)			Specificity (%)	
*(Feature Sets)*	*(All)*	*(MRMR)*	*(CNN)*	*(All)*	*(MRMR)*	*(CNN)*	*(All)*	*(MRMR)*	*(CNN)*
**SVM**	97.29	97.08	96.46	97.29	97.08	96.46	99.61	99.58	99.49
**Wide-NN**	96.67	95.83	97.29	96.67	95.83	97.29	99.52	99.40	99.61
**Medium-NN**	96.46	95.42	95.21	96.46	95.42	95.21	99.49	99.35	99.32
**LDA**	96.04	93.96	96.04	96.04	93.96	96.04	99.43	99.14	99.43
**Ensemble Bagged Trees**	96.04	96.25	96.67	96.04	96.25	96.67	99.43	99.46	99.52
**K-NN**	95.83	95.42	96.25	95.83	95.42	96.25	99.40	99.35	99.46
**Ensemble Boosted Trees**	96.46	95.83	95.83	96.46	95.83	95.83	99.49	99.40	99.40
**Naive Bayes**	94.58	94.58	95.63	94.58	94.58	95.63	99.23	99.23	99.38

## Data Availability

Data underlying this research is owned and managed by Ulster University, UK, in accordance with institutional data management policies ensuring ethical, legal, and professional compliance for access.
